# Ethnic Disparities for Survival and Mortality in New Zealand Patients With Head and Neck Cancer

**DOI:** 10.1001/jamanetworkopen.2024.13004

**Published:** 2024-06-04

**Authors:** Abigail Weaver, Sarah Twine, Melissa Bather, Andrew Dowley, Cristian M. Slough

**Affiliations:** 1 Otolaryngology Department, Hawkes Bay District Health Board, Hastings Hospital, Hasting, New Zealand; 2 University of Auckland, Auckland Central Business District, Auckland, New Zealand

## Abstract

**Importance:**

It is essential to identify inequitable cancer care for ethnic minority groups, which may allow policy change associated with improved survival and decreased mortality and morbidity.

**Objective:**

To investigate ethnic disparities in survival and mortality among New Zealand (NZ) patients with head and neck cancer (HNC) and the association of other variables, including socioeconomic status, tumor stage, and age at diagnosis, with survival rates.

**Design, Setting, and Participants:**

This retrospective cohort study was conducted among NZ patients diagnosed with specific HNCs from 2010 to 2020. Anonymized data were obtained from the NZ Cancer Registry, including patients diagnosed from *International Statistical Classification of Diseases and Related Health Problems, Tenth Revision *(*ICD-10*) codes C00-C14 and C30-C32. Data were analyzed from July 2020 through January 2024.

**Main Outcomes and Measures:**

Censored Kaplan-Meier estimates were used to analyze survival distribution. Cox regression models were used to estimate the association of age, tumor stage at diagnosis, and socioeconomic status with survival rates. Age-standardized mortality rates were assessed.

**Results:**

Among 6593 patients with HNCs (4590 males [69.6%]; 4187 patients aged 51-75 years [63.5%]), there were 706 Māori individuals (10.7%) and 5887 individuals with other ethnicity (89.3%), including 4327 NZ European individuals (65.6%; defined as New Zealanders of European descent). Māori individuals had a decreased survival proportion at all years after diagnosis compared with individuals with other ethnicity (eg, 66.1% [95% CI, 62.6%% to 69.8%] vs 71.2% [95% CI, 70.0% to 72.4%] at 2 years). At 1 year after diagnosis, Māori individuals did not have a significantly increased mortality rate compared with 5795 individuals with other ethnicity with data (193 deaths [27.3%] vs 1400 deaths [24.2%]; *P* = .06), but the rate was significantly increased at 5 years after diagnosis (277 deaths [39.3%] vs 2034 deaths [35.1%]; *P* = .03); there was greater disparity compared with NZ European individuals (1 year: 969 deaths [22.4%]; *P* = .003; 5 years: 1441 deaths [33.3%]; *P* = .002). There were persistent age-adjusted mortality rate disparities: 40.1% (95% CI, −25.9% to 71.2%) for Māori individuals and 18.8% (95% CI, −15.4% to 24.4%) for individuals with other ethnicity. Māori individuals were diagnosed at a mean age of 58.0 years (95% CI, 57.1-59.1 years) vs 64.3 years. (95% CI, 64.0-64.7 years) for individuals with other ethnicity, or 5 to 7 years younger, and died at mean age of 63.5 years (95% CI, 62.0-64.9 years) compared with 72.3 years (95% CI, 71.8-72.9 years) for individuals with other ethnicity, or 7 to 10 years earlier. Māori individuals presented with proportionally more advanced disease (only localized disease, 102 patients [14.5%; 95% CI, 12.0%-17.4%] vs 1413 patients [24.0%; 95% CI, 22.9%-25.1%]; *P* < .001) and showed an increase in regional lymph nodes (276 patients [39.1%; 95% CI, 35.5%-42.9%] vs 1796 patients [30.5%; 95% CI, 29.3%-31.8%]; *P* < .001) at diagnosis compared with individuals with other ethnicity. Socioeconomic status was not associated with survival.

**Conclusions and Relevance:**

This study found that Māori individuals experienced worse survival outcomes and greater mortality rates from HNC in NZ and presented with more advanced disease at a younger age. These findings suggest the need for further research to alleviate these disparities, highlight the importance of research into minority populations with HNC globally, and may encourage equity research for all cancers.

## Key Points

**Question:**

Are there ethnic disparities for survival and mortality in New Zealand patients with head and neck cancer?

**Findings:**

In this cohort study of 6593 patients with head and neck cancer, Māori patients had decreased survival proportions at all years after diagnosis compared with patients with other ethnicity; age-standardized mortality rates were 40.1% for Māori and 18.8% for patients with other ethnicity.

**Meaning:**

These findings suggest that policy change may be required given that Māori individuals experienced worse survival outcomes and greater mortality rates from head and neck cancer in New Zealand.

## Teaser

This cohort study investigates disparities in survival and mortality between Māori patients and patients from other ethnic groups with head and neck cancer in New Zealand.

## Introduction

Health inequity for Māori individuals, the Indigenous, minority population of New Zealand (NZ), is evident in NZ literature, identifying poorer health outcomes with increased morbidity and mortality, poorer access to health care, and decreased quality of care in this population. These inequities persist in oncology care. Māori patients were less likely to receive chemotherapy and more likely to have delayed initiation of chemotherapy.^1,2^ Māori populations have an approximately 20% increased risk of cancer and 2 times the cancer mortality compared with populations with other ethnicity.^3^ Māori populations have an increased risk of mortality for the most commonly occurring cancers in NZ.^4^ This substantial health gap has been improving through governmental policy focusing on Indigenous health^4^

Head and neck cancer (HNC) is the ninth most common cancer globally and causes significant morbidity and mortality for patients.^5^ NZ has one of the highest incidences of HNC, with 500 to 550 new cases annually in a population of 5 million people.^6^ Given the high prevalence of HNC in our population, identification of inequities in provision of health care is crucial for appropriate resource management. Similar studies have been done in US populations. US literature has identified poorer survival rates for African American patients with HNC compared with White populations.^7,8^ A meta-analysis^7^ found that African American patients had statistically significant poorer overall survival compared with White patients, with a hazard ratio (HR) of 1.27 after multivariate analysis.

Despite identified inequities in HNC internationally, there is a paucity of local literature. The NZ Ministry of Health has reported HNC trends from 1991 to 2004, which identify a statistically significant greater excess mortality for Māori populations with HNC compared with populations with other ethnicity, with an excess mortality rate ratio of 1.37.^9^ Comparatively, a study in 2014 to 2019 in Waitemata, NZ, analyzed 186 oral cavity and oropharynx squamous cell carcinomas and found no mortality rate disparities for Māori compared with populations with other ethnicity. Due to the small study population, outcomes were not statistically significant, and a larger multicenter study was recommended.^10^

This cohort study investigates a large population of patients with HNCs in NZ from 2010 to 2020, with the aim to identify survival and mortality rate disparities for Māori populations diagnosed with HNC. Furthermore, we used regression models to analyze the association of stage at diagnosis, socioeconomic status, and age at diagnosis with survival rate inequities. Identification of inequities should be further investigated to improve health outcomes for Māori populations.

## Methods

This cohort study was deemed exempt from Health and Disability Ethics Committees and informed consent requirements by the Ministry of Health, NZ government because this was an observational study with no access to confidential information. The manuscript is reported following the Strengthening the Reporting of Observational Studies in Epidemiology (STROBE) reporting guideline.

Data were obtained from the NZ Cancer Registry, a national registry of all primary malignant neoplasms, excluding squamous and basal cell skin cancers. The registry provided anonymized datasets from 2010 to 2020. *International Statistical Classification of Diseases and Related Health Problems, Tenth Revision *(*ICD-10*) cancer codes C00-C14 and C30-C32, representing cancer of the nasal cavity, middle ear, accessory sinuses and larynx, lip, oral cavity, pharynx, and larynx, were included. All ages at diagnosis were included. Ethnicity was derived from self-reported data from the National Health Index database; patients could self-report any ethnicity. These cohorts included 706 Māori individuals and 4327 NZ European individuals (defined as New Zealanders of European descent) within an overall group with other ethnicity of 5887 individuals.

The database included date of diagnosis, date of death, age at diagnosis, tumor stage at diagnosis, and domicile code. The NZ Cancer Registry uses the Surveillance, Epidemiology, and End Results (SEER) General Summary Staging System to record tumor staging. This separates tumor burden into 6 categories: distant metastases, regional lymph nodes, invasion of adjacent tissue or organ, localized to organ of origin, and unknown. Socioeconomic status levels were identified using the NZ Index of Deprivation scale adapted from the domicile code indicating the patient’s address. This scale is an area-based measure of socioeconomic status measuring the relative deprivation of small areas, with 1 being the least deprived and 10 being the most deprived. A retrospective review of this dataset was performed investigating the association of ethnicity with 1- and 5-year mortality for patients with HNC in NZ.

Defined ethnic subgroups included Māori, other ethnicity, and NZ European populations. The other ethnicity subgroup included all known ethnicities who did not identify as Māori: African, Asian, European (ie, immigrated from Europe), NZ European (ie, born in NZ with European ancestry), and Pacific. To further exclude the association of minority outcomes with health, an additional NZ European group was analyzed for comparison with the Māori population (it is best to exclude other minority populations from the other ethnicity population because poorer outcomes for minority groups, such as Pacific Islander individuals, minimize disparities between NZ European and Māori populations). Māori, other ethnicity, and NZ European populations were analyzed to identify mortality rates and tumor extent at diagnosis. Further data compared Māori and populations with other ethnicity.

### Statistical Analysis

R statistical software version 4.1.3 (R Project for Statistical Computing) was used for data analysis. Survival and mortality comparisons were based on complete years survived after diagnosis. Kaplan-Meier estimates were used to identify survival distribution with censoring. Mortality rates at 1 and 5 years after diagnosis were identified for Māori, other ethnicity, and NZ European populations. Mortality rate was identified using the percentage of the ethnic population who had died within or equal to that year after diagnosis, only patients who had a diagnosis of 5 years or longer were included in 5-year rates. We did not include 10-year mortality rate owing to likely confounding factors for which we could not account, including age, comorbidities, and cause of death. Cox proportional hazard models were used to investigate the association of age, decile (an area-based measure of socioeconomic status separating the population into 10 groups, with decile 1 being the least deprived and decile 10 being the most deprived), and tumor stage with survival rates for Māori and populations with other ethnicity. We fitted 3 general linear models using 1-, 5-, and 10-year survival after diagnosis as response variables. Explanatory variables used were year of diagnosis, SEER disease extent, age at diagnosis, decile, and ethnicity. All combinations of explanatory variables were investigated for each response and the best models selected. The significance level was *P* < .05. The Welch *t* test was used to investigate the difference in mean age of diagnosis and death for other ethnicity and Māori populations. Data were analyzed from July 2020 through January 2024.

## Results

A total of 6593 people (4590 males [69.6%] and 2003 females [30.4%]; 4187 patients aged 51-75 years [63.5%]) were diagnosed with nasal cavity, middle ear, accessory sinuses and larynx, lip, oral cavity, pharynx, and larynx cancer in NZ between 2010 and 2020. These patients included 706 Māori individuals (10.7%) and 4327 NZ European individuals (65.6%) within an overall population of 5887 individuals with other ethnicity (89.3%). There were 476 Māori and 3711 individuals with other ethnicity aged 51 to 75 years. Demographics are displayed in Table 1. The registry included unknown ethnicities, which were excluded from analysis.

**Table 1.** Demographics of Study Population

Demographic characteristic	Patients, No. (%)			
	Total (N = 6593)	Māori (n = 706)	Other ethnicity	
			Overall (n = 5887)	NZ European (n = 4327)
Sex^a^				
Male	4590 (69.6)	496 (7.5)	4094 (62.1)	3016 (45.7)
Female	2003 (30.4)	210 (3.2)	1793 (27.2)	1311 (19.9)
Age, y				
<31	133 (2.0)	18 (0.3)	115 (1.7)	57 (8.6)
31-50	926 (14.0)	156 (2.4)	770 (11.7)	496 (7.5)
51-75	4187 (63.5)	476 (7.2)	3711 (56.3)	2712 (41.1)
>75	1347 (20.4)	56 (0.8)	1291 (19.6)	1062 (16.1)

Abbreviation: NZ, New Zealand.

^a^ Sex was self-identified using the National Health Index.

### Incidence Rates

**Figure 1.**
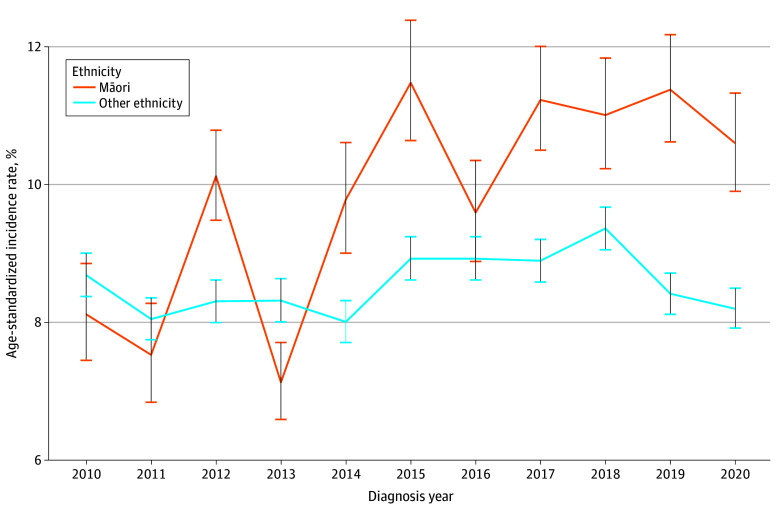
Age-Standardized Incidence Rates of Head and Neck Cancer Whiskers indicate 95% CIs.

From 2012 to 2020, excluding 2013 and 2016, there was a statistically significant difference in the incidence rate of HNC between Māori and individuals with other ethnicity. In these years, Māori individuals had a statistically higher incidence rate than individuals with other ethnicity, except for 2013, when individuals with other ethnicity had a higher incidence rate. The higher incidence rate for Māori individuals has become more apparent since 2014 (Figure 1).

### Survival Rates

**Figure 2.**
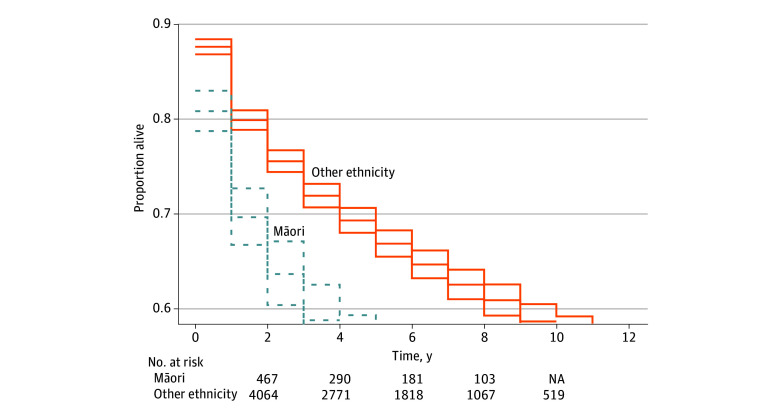
Censored Kaplan-Meier Survival Distribution NA indicates not applicable. Upper and lower lines indicate 95% CIs.

Kaplan-Meier estimates display survival distribution comparing Māori and populations with other ethnicity (Figure 2). Māori individuals had a decreased survival proportion at all years after diagnosis; for example, the survival rate at 2 years after diagnosis was 66.1% (95% CI, 62.6%-69.8%) for Māori and 71.2% (95% CI, 70.0%-72.4%) for individuals with other ethnicity.

### Mortality Rates

The rate of mortality was identified at 1 and 5 years for 706 Māori individuals and 5795 individuals with other ethnicity with data, including NZ European individuals. At 1 year after diagnosis, 193 Māori individuals had died (27.3%) compared with 1400 individuals with other ethnicity (24.2%), which was not a significantly greater mortality rate for Māori individuals (*P* = .06). A significant difference in mortality rate was identified at 5 years after diagnosis, with 277 deaths among Māori individuals (39.3%) vs 2034 deaths among individuals with other ethnicity (35.1%), a difference in mortality rate of 4.2 percentage points (*P* = .03). Age-standardized mortality rates were 40.1% (95% CI, −25.9% to 71.2%) for Māori individuals and 18.8% (95% CI, −15.4% to 24.4%) for individuals with other ethnicity, thus showing a statistically significant difference. NZ European individuals had the lowest mortality rate compared with individuals with other ethnicity and Māori individuals at 1 and 5 years (Table 2). Mortality rates for Māori vs NZ European individuals were 27.3% vs 969 deaths (22.4%) at 1 year (*P* = .003) and 39.3% vs 1441 deaths (33.3%) at 5 years (*P* = .002), for increased rates among Māori individuals at 1 and 5 years after diagnosis of 5.0 and 6.0 percentage points, respectively, compared with NZ European individuals (Table 2). Using Cox regression modeling adjusting for age, sex, and year of diagnosis, Māori individuals had a mortality HR of 1.6 (95% CI, 1.4-1.8) compared with individuals with other ethnicity.

**Table 2.** Mortality Rate Disparities

Time since diagnosis, y	Mortality rate, No. (%)			Māori vs other ethnicity rate increase, percentage points	*P* value	Māori vs NZ European rate increase, percentage points	*P* value
	Māori (n = 706)	Other ethnicity					
		Overall (n = 5795)	NZ European (n = 4327)				
1	193 (27.3)	1400 (24.2)	969 (22.4)	3.2	.06	5.0	.003
5	277 (39.3)	2034 (35.1)	1441 (33.3)	4.2	.03	6.0	.002

Abbreviation: NZ, New Zealand.

### Age

Further Cox regression models were used to investigate the association of age and decile with survival in Māori and populations with other ethnicity. Age at diagnosis was the only factor associated with survival for Māori and populations with other ethnicity, with a linear association between increasing age at diagnosis and decreasing survival rates (eTable 1 in Supplement 1). Welch 2-sample *t* tests identified that Māori individuals were diagnosed at younger ages. The mean age of diagnosis was 58.0 years (95% CI, 57.1-59.1 years) for Māori individuals compared with 64.3 years (95% CI, 64.0-64.7 years) for individuals with other ethnicity; 95% CIs show that the mean age of diagnosis was 5 to 7 years younger among Māori vs individuals with other ethnicity. Of individuals who died, Māori individuals died at mean age of 63.5 years (95% CI, 62.0-64.9 years) compared with 72.3 years (95% CI, 71.8-72.9 years) for individuals with other ethnicity; 95% CIs show that the mean age at death was 7 to 10 years earlier among Māori than individuals with other ethnicity (eTable 1 in Supplement 1).

### Tumor Extent at Diagnosis

To determine the proportion of patients by level of disease extent, each ethnic subgroup was separated and the percentage of each disease extent was identified. Māori individuals were proportionally more likely to be diagnosed with higher disease staging compared with individuals with other ethnicity, with greater disparities compared with NZ European individuals. Presentation with only localized disease with no locoregional or distant spread occurred among 1413 patients with other ethnicity (24.0%; 95% CI, 22.9%-25.1%) compared with 102 Māori patients (14.5%; 95% CI, 12.0%-17.4%) (*P* < .001). Māori patients were proportionally more likely to be diagnosed with regional lymph node involvement compared with patients with other ethnicity (276 patients [39.1%; 95% CI, 35.5%-42.9%] vs 1796 patients [30.5%; 95% CI, 29.3%-31.8%]; *P* < .001). NZ European patients were proportionally more likely to be diagnosed with tumors localized to the organ of origin compared with Māori patients (1090 patients [25.2%; 95% CI, 23.9%-26.5%] vs 102 patients [14.5%; 95% CI, 12.0%-17.4%]; *P* < .001). Māori patients were proportionally more likely to present with regional lymph node involvement compared with NZ European patients (1307 patients [30.2%; 95% CI, 28.8%-31.6%]; *P* < .001). Disparities between Māori patients and patients with other ethnicity or NZ European patients for tumors localized to organ of origin and regional lymph nodes were statistically significant (eTable 2 in Supplement 1).

Regression models used local disease as a baseline variant and compared it with all other categories for extent of disease. A linear association between increased disease progression at diagnosis and survival was identified by HRs. Māori patients had greater HRs at all disease extents compared with patients with other ethnicity, indicating poorer survival when presenting with comparable tumor spread. These differences were not statistically significant given that 95% CIs for Māori and patients with other ethnicity overlapped owing to the smaller Māori population; however, HR point estimates were consistently higher for Māori vs patients with other ethnicity. Māori patients had an HR of 3.26 (95% CI, 1.90-5.61; *P* < .001) for regional lymph node involvement and 7.92 (95% CI, 4.37-14.40; *P* < .001) for distant metastasis compared with localized disease. Patients with other ethnicity had lower HRs of 2.47 (95% CI, 2.15-2.83; *P* < .001) and 5.63 (95% CI, 4.74-6.68; *P* < .001) for regional lymph nodes and distant metastasis, respectively, compared with localized disease. Results summarized in Table 3.

**Table 3.** Risk of Mortality by Advancing Tumor Extent at Diagnosis

Tumor extent at diagnosis	Mortality for Māori, HR (95%CI)^a^	*P* value	Mortality for other ethnicity, HR (95%CI)^a^	*P* value
Invasion into adjacent tissue or origin	2.83 (1.34-5.96)	.006	2.13 (1.72-2.64)	<.001
Regional lymph nodes	3.26 (1.90-5.61)	<.001	2.47 (2.15-2.83)	<.001
Distant metastasis	7.92 (4.37-14.40)	<.001	5.63 (4.74-6.68)	<.001
Unknown	3.58 (2.09-6.14)	NA	2.44 (2.14-2.78)	NA

Abbreviations: HR, hazard ratio; NA, not applicable.

^a^ Reference was local disease.

### Socioeconomic Status and Date of Diagnosis

Decile and date of diagnosis had no association with survival rates for Māori or patients with other ethnicity. Cox regression analysis using survival rate was used to compute Cox proportional hazard regression models for each decile for Māori and populations with other ethnicity. At each decile for both ethnicities, there was no association between decile and survival rate (eTable 3 in Supplement 1).

## Discussion

The World Health Organization describes health inequities as “systematic differences in the health status of different population groups.”^11^ Health inequities have continually been identified within the NZ health care system, contributing to a large socioeconomic burden. Health inequities are avoidable and preventable disparities between subgroups within a population that should be alleviated with policy change and resource distribution.^12^ This cohort study highlights inequities for Māori patients with HNC. From 2012 to 2020, excluding 2013 and 2016, there was a statistically significantly increased incidence rate of HNC for Māori compared with populations with other ethnicity.

### Survival and Mortality Rate

Kaplan-Meier estimates showed a statistically significant pattern of decreased survival at all years after diagnosis for Māori compared with patients with other ethnicity. Māori patients had a statistically significant greater age-standardized mortality rate at 40.1% compared with patients with other ethnicity at 18.8%. Mortality rates prior to age standardization were 5.0 and 6.0 percentage points greater for Māori than NZ European individuals at 1 and 5 years after diagnosis, respectively. Mortality rates for patients with other ethnicity were less than those for Māori patients but greater than those for NZ European patients owing to the inclusion of other minority ethnicities, such as Pacific populations.

### Age

Age was the only variable associated with survival for both Māori and populations with other ethnicity, with an inverse proportional association between increasing age at diagnosis and decreased survival. With increasing age, there is greater comorbidity and frailty and consequently fewer treatment options. Māori patients’ mean age of diagnosis was 5 to 7 years younger than that of patients with other ethnicity, and their mean age of death was 7 to 10 years earlier. This suggests that NZ Māori patients have an increased loss of working years owing to morbidity and mortality of HNC. The mean age of diagnosis for Māori patients was 7 years younger than retirement age (65 years), while the mean age of diagnosis of patients with other ethnicity was less than 1 year younger than retirement age. This may have a perpetuating association with poverty and is a large economic loss for the population.^13^

### Tumor Burden at Diagnosis

Proportionally, Māori patients presented with more advanced disease, with a statistically significant proportion of less localized disease and greater regional lymph nodes at diagnosis compared with patients with other ethnicity and NZ European patients. A more advanced tumor at diagnosis is associated with poorer survival. Māori patients had poorer survival at all tumor stages compared with local disease, with Māori patients having greater mean HRs than patients with other ethnicity, although this difference was not statistically significant.

Limited access to primary health care, especially in rural areas where there is a higher proportion of Māori individuals in the population, may contribute to these late presentations. A higher proportion of Māori individuals (32.8%) live in rural areas compared with NZ European individuals (26.3%).^14^ Access to general practitioners (GPs) for referrals or investigations may be further reduced, with 20.6% of the GP workforce working in rural areas.^15^ Access to primary health may also be hindered by transportation. In 2020, greater than 6% of Māori adults reported that they were unable to visit a GP due to lack of transport.^16^

Further NZ literature has identified survival disparities for cancers between Māori and populations with other ethnicity due to poorer access to early diagnosis and treatment.^1,3,4,17,18,19^ A study^20^ found that Māori patients were more likely to be admitted to tertiary care without interventional cardiology services, receiving fewer investigations and interventions, which was associated with poorer outcomes. Access to care in NZ has geographic disparities, often requiring patients to travel out of region for radiation facilities and surgical subspecialists. This represents another barrier for Māori individuals to access necessary care, exacerbating health disparities.

Access disparities are also evident within dental care. Dental examinations help identify early-stage oral cancer, but 73% of Māori individuals self-reported visiting dental health workers only for acute dental problems or not at all within the last year compared with 50.9% of individuals with other ethnicity.^21^ With access to health care playing a role in a delayed diagnosis for patients with HNC, it is important to note that Māori adults were 1.5 times more likely to report cost as a barrier to seeing a primary care physician vs adults with other ethnicity.^22^

### Non–Statistically Significant Variables

Socioeconomic status was not associated with survival rate, regardless of ethnicity. NZ operates on a public health model. with prioritization and auditing of cancer diagnosis and treatment to ensure efficacy. Public tertiary medical care is free in NZ regardless of health insurance status. Protocol suggests that the time from referral to first specialist appointment should be 2 weeks, with the initiation of treatment in 6 weeks. This may explain why socioeconomic status was not associated with survival rates. However, it is important to note that the NZ Index of Deprivation system is based on a person’s address not individual socioeconomic status.

### Strengths and Limitations

This study has several strengths. It used 10 years of national data, including all recorded specific HNCs. The Ministry of Health data included variables of ethnicity, age, domicile, and tumor stage, allowing for standardization and regression models to estimate the association of these variables with overall survival rates.

This study also has limitations. Within the other ethnicity demographic, there were multiple ethnicities, some of which were unknown and had to be excluded from our study. Important variables, including, smoking, alcohol consumption, rural location, regular GP, comorbidities, treatment modalities, tumor pathology, p16 status, and human papillomavirus vaccination status, are not recorded in the NZ cancer registry; consequently, the association of these factors with survival could not be interpreted within this study.

Māori populations have disproportionately increased rates of multiple modifiable health determinants that are associated with increased risk of HNC,^22^ with increased rates of smoking and alcohol consumption in the NZ census data.^22^ This is a limitation given that we do not have smoking or alcohol data available to determine the association of these factors with inequitable outcomes. Another contributor to differing prognosis includes the histology of HNC. This is not disclosed in the cancer registry data; therefore, its association with survival rates could not be determined. Additionally, we were unable to analyze disease-specific mortality given that the data do not directly comment on whether the patient died from the cancer.

## Conclusions

This cohort study highlights disparities in survival and mortality rates in NZ patients with HNC, finding that Māori patients had higher mortality rates and poorer survival compared with patients with other ethnicity after an HNC diagnosis. Māori patients had a statistically significantly increased age-standardized mortality rate compared with patients with other ethnicity. Additionally, Māori patients were diagnosed and died at younger ages than patients with other ethnicity. Māori patients were also proportionately more likely to present with a more advanced extent of tumor. Using SEER staging, we found that Māori patients presented with proportionally less local disease and greater regional lymph nodes compared with patients with other ethnicity and NZ European patients. Socioeconomic status was not associated with survival. This study has identified inequities and analyzed potential factors associated with this disparity and may motivate further research into the cause of these inequities. Results of this study may identify outcomes associated with inequitable care identified in literature discussed previously. Further research is crucial and may allow for intervention and policy change to alleviate these disparities. This further work should focus on the association of other variables (including time from diagnostic procedure to treatment, treatment modality, modifiable health factors, comorbidities, viral status, and histology) with these inequitable mortality and survival rates and use qualitative surveys to understand factors associated with treatment decisions. Hence further study may be warranted if inequities are accounted for by these mediating variables, and targeted health promotion and policy may be implemented. This study highlights not only the importance of research into ethnic minority populations with HNC globally, but may also encourage equity research for all cancer presentations.

## Notes

Supplement 1. **eTable 1.** Age at Diagnosis and Death **eTable 2.** Disease Extent **eTable 3.** Decile and Date of Diagnosis

Supplement 2.
Data Sharing Statement
