# Missing Sentence in Methods

**DOI:** 10.1001/jamanetworkopen.2025.40435

**Published:** 2025-10-03

In the Original Investigation titled “Ethnic Disparities for Survival and Mortality in New Zealand Patients With Head and Neck Cancer,”^1^ published June 4, 2024, a sentence explaining that comparisons were based on complete years survived after diagnosis was missing. The article has been corrected.^1^
