# Supplementary material for: Ethnic Disparities for Survival and Mortality in New Zealand Patients With Head and Neck Cancer
Source: JAMA Netw Open. 2024 Jun 4;7(6):e2413004. doi: 10.1001/jamanetworkopen.2024.13004 (PMC11151153; doi:10.1001/jamanetworkopen.2024.13004)
Supplement: Supplement 1. — eTable 1. Age at Diagnosis and Death eTable 2. Disease Extent eTable 3. Decile and Date of Diagnosis [file jamanetwopen-e2413004-s001.pdf]

Supplemental Online Content

Weaver A, Twine S, Bather M, Dowley A, Slough CM. Ethnic disparities for survival and mortality in New Zealand patients with head and neck cancer. *JAMA Netw Open*. 2024;7(6):e2413004. doi:10.1001/jamanetworkopen.2024.13004

eTable 1. Age at Diagnosis and Death
eTable 2. Disease Extent
eTable 3. Decile and Date of Diagnosis

This supplemental material has been provided by the authors to give readers additional information about their work.

© 2024 Weaver A et al. *JAMA Network Open*.

eTable 1. Age at Diagnosis and Death

	Non-Māori	Māori	Difference between Māori and non-Māori
Mean age at Diagnosis<br>- Unit: Years	64.3 (95% CI, 64.0-64.7)	58.0 (95% CI, 57.1-59.1)	Māori: 5-7 younger
Mean age at Death<br>- Uinit: Years	72.3 (95% CI, 71.8-72.9)	63.5 (95% CI,62.0-64.9)	Māori: 7-10 younger

© 2024 Weaver A et al. *JAMA Network Open*.

eTable 2. Disease Extent

	Disease localized to organ of origin	Regional lymph node involvement	Distant Metastasis	Unknown
Non-Māori , Including NZ European, Excluding unknown n = 5887	24% (CI 95% 22.9-25.1) n=1413	30.5% (CI 95% 29.3-31.8) n=1796	6.0% (CI 95% 5.4-6.6) n=353	34.4% (CI 95% 33.1-35.6) n=2025
NZ European n = 4327	25.2% (CI 95% 23.9-26.5) n=1090	30.2% (CI 95% 28.8-31.6) n=1307	5.4% (CI 95% 4.8 - 6.2) n=234	33.9% (CI 95% 32.5%- 35.4) n=1467
Māori n=706	14.5% (CI 95% 12.0-17.4) n=102	39.1% (CI 95% 35.5 - 42.9) n=276	7.7% (CI 95% 5.9 - 10.0) n=54	34.4% (CI 95% 30.9-38.2 n=243

© 2024 Weaver A et al. *JAMA Network Open*.

eTable 3. Decile and Date of Diagnosis

	Māori Survival based on covariates - Hazard Ratio	P-value
Decile 2	1.03	0.95
Decile 3	0.43	0.16
Decile 4	0.83	0.67
Decile 5	0.77	0.55
Decile 6	1.20	0.66
Decile 7	0.81	0.61
Decile 8	1.59	0.24
Decile 9	1.40	0.37
Decile 10	1.25	0.43

© 2024 Weaver A et al. *JAMA Network Open*.
