# Supplementary material for: Ethnic Disparities for Survival and Mortality in New Zealand Patients With Head and Neck Cancer
Source: JAMA Netw Open. 2024 Jun 4;7(6):e2413004. doi: 10.1001/jamanetworkopen.2024.13004 (PMC11151153; doi:10.1001/jamanetworkopen.2024.13004)
Supplement: Supplement 2. — Data Sharing Statement [file jamanetwopen-e2413004-s002.pdf]

Data Sharing Statement

Weaver. Ethnic Disparities for Survival and Mortality in New Zealand Patients With Head and Neck Cancer. *JAMA Netw Open*. Published online May 23, 2024. doi:10.1001/jamanetworkopen.2024.13004

Data
Data available: No

Additional Information
Explanation for why data not available: Health Board Protected data. The data can be made available if so desired. Please contact corresponding author.
